# Effects of Wii Fit-based Exercises versus proprioceptive training on balance and fall risk in patients with diabetic neuropathy

**DOI:** 10.12669/pjms.41.2.8775

**Published:** 2025-02

**Authors:** Syed Shakil ur Rehman, Danish Hassan, Maryam Ikram, Mehwish Ikram

**Affiliations:** 1 Syed Shakil ur Rehman, PhD (PT). Faculty of Rehabilitation and Allied Health Sciences, Riphah International University, Lahore, Pakistan; 2 Danish Hassan, PhD (PT). Faculty of Rehabilitation and Allied Health Sciences, Riphah International University, Lahore, Pakistan; 3 Maryam Ikram, MS (PT) Faculty of Rehabilitation and Allied Health Sciences, Riphah International University, Lahore, Pakistan; 4 Mehwish Ikram, MS (PT) Faculty of Rehabilitation and Allied Health Sciences, Riphah International University, Lahore, Pakistan

**Keywords:** Physical Activities, Proprioception, Exercise, Accidental falls, Physical Therapy Techniques

## Abstract

**Objective:**

To determine the effects of Wii Fit-based exercises and proprioceptive training on balance and fall risk in diabetic neuropathy patients.

**Methods:**

This randomized clinical trial was conducted in Riphah Rehabilitation Centre, Lahore, between March 2022 to August 2022 (duration six months). Thirty-four participants were included, twenty males and fourteen females. Participants were randomly allocated into two groups. Group-A received Wii Fit-based exercises thrice a week for six weeks and Group-B received Proprioceptive training thrice a week for six weeks. Pre and post-measurements of balance and risk fall were assessed through Berg Balance Scale (BBS), Modified Fall Efficacy Scale (MFES), and Timed Up and Go test (TUG).

**Results:**

Thirty-four participants (20 males and 14 females) were allocated in each group, with a mean age of 62±9.21. The normality test (Shaphiro-Wilk Test, p>0.05) of BBS, MFES, and TUG summarized that data was normally distributed. Six weeks of treatment sessions have shown no significant difference between both groups (p>0.05). However, the within-group comparisons showed significant results that indicate the clinical effects of both treatments (p<0.05).

**Conclusion:**

It was concluded that there was no difference between Wii Fit exercises and proprioceptive training on balance and risk of falls in diabetic neuropathic patients.

***Trial Registration Number:*** This study was registered at ClinicalTrials.gov ID: NCT05282602 on date 16/03/2022.

## INTRODUCTION

Diabetic Neuropathy (DN) leads to postural instability, increase risk of falls, and inability to perform daily life activities (ADL).^1^ Neuropathy affects long fibres and symptoms seem on the toes and feet and gradually symptoms spread to the hands and legs, this causes paraesthesia; Loss of Protective Sensation (LOPS) and balance problems.^2^ Patients with diabetic peripheral neuropathy (DPN) suffer from physiological instability that causes excessive postural sway and postural imbalance. The risk of falls increases because great muscle effort is required to maintain the balance of the body.^3^,^4^ Patients with DPN may also have foot muscle dysfunction, leading to abnormal gait, compromise of daily activities, and increased risk of falls.^5^

Nintendo Wii exergames have been used worldwide for rehabilitation. Virtual reality games produce positive effects in improving static/dynamic balance, posture, daily living activities and reducing falls.^6^ Nintendo Wii (virtual reality) based technique control movement of the limbs with a hand controller and balance board. Wii-based exercise games are installed in the game space.^7^ Wii Fit balance board can improve the mediolateral and anterior-posterior movement of the body.^8^ The Nintendo Wii balance board detects vibrations and perturbations that detect the patient’s pressure centre.^9^ Virtual reality-based rehabilitation is a recent technology that challenges the vestibular system and proprioceptors. Wii Fit is software for strengthening the core muscles and improving the endurance of muscles. Wii Fit is inexpensive as compared to other virtual reality exergaming tools and easy to use at home.^10^ Proprioception training has better results in improving the balance and motor control of the lower extremities.^11^

A few studies were available on the use of Wii fit-based exercises on balance and risk of falls in diabetic neuropathic patients but found no studies in comparison with proprioceptive exercises.^12^-^16^ The rationale of this study was to determine the effects of two different trainings on diabetic neuropathic patients (balance and fall risk which showed better results and can be used in clinics for rehabilitation.The purpose of this study was to find out the effect of both techniques in diabetic neuropathy patients with poor balance and fall risk. It was hypothesized that there was a difference in Wii fit-based exercises and proprioceptive training on balance and fall risk in diabetic neuropathic patients.

## METHODS

It was a randomized clinical trial registered in ClinicalTrials.govt with reference number NCT05282602 on dated March 16, 2022. The study was conducted at Riphah Rehabilitation Center (Clinic), Lahore, Pakistan.

### Ethical Approval:

The study was completed in six months, starting from March 2022 to August 2022 after the approval of the ethical committee of RCRS & Allied Health Sciences, Riphah International University, Lahore with a reference number of REC/RCR & AHS/22/0201, dated January 28, 2021.

The sample size was calculated from the results of a previous study using values of the mean difference of the Numerical Pain Rating Scale (NPRS). After the addition of a 10% attrition rate, a sample size of thirty-four was calculated by using the online epitool calculator (5% variance and 95% confidence interval).^14^ Non-probability convenience sampling technique was used to recruit the sample. Participants 40-80 years of age; Douleur Neuropathique en four questionnaire (DN4>4) with controlled diabetes^17^, self-reported ability to walk and no cognitive impairment (MMSE>24) were included in the study. Neurological diseases such as Guillain Barre Syndrome (GBS), dementia delusions, Parkinson’s disease, Spinal Cord Injury (SCI), or acute, sub-acute and chronic stroke; patients with other neurological diseases like dementia, visual impairment, and other diseases through which patients unable to walk and high fall risk were excluded.

After the screening of forty patients based on inclusion and exclusion criteria, 34 participants were selected for the research. Consent papers were signed by all participants for research, then participants were randomly allocated into two groups (A and B) using a lottery method by a physiotherapist not involved in the study plan. A (for Group-A) and B (for Group-B) letters were written on the folded slips of paper, those slips were stored in a box. The participants were allocated randomly based on selected folded slips by a physiotherapist without looking at the slips (added consecutively). Group-A received Wii Fit-based exercises and Group-B received Proprioceptive training exercises. The outcome assessor was blinded from the allocation of the treatment protocol.

### Group-A:

Group-A participants received Wii Fit-based exercises thrice a week for six weeks. Wii Fit game-based exercises included penguin slide, soccer heading, tilt table, and ski jump. Wii Fit exercises are virtual-based exercises and more challenging than any other exercises. Virtual exercises were explained to the participant and a detailed workout was discussed. Assistance was given, accountability, monitoring, follow-ups, and every detail of exercises were recorded. Each exercise was performed for ten minutes under supervision on Wii Fit, five to six sets with a thirty-second rest between each set, and a rest of one minute between each exercise. The total duration was of fifty minutes including five minutes of warm-up sessions (lower limb stretching).^15^

### Group-B:

Group-B participants received Proprioceptive training exercises thrice a week for six weeks. Proprioceptive training exercises included toe walking, heel walking, cross-body leg swings, and partial squat (in sitting). Each exercise was performed for ten minutes under supervision, five sets of each exercise with a rest of one minute between each exercise. The total duration was of fifty minutes including five minutes of warm-up sessions (lower limb stretching).^16^ Patients with diabetic neuropathy associated with diabetes mellitus were included n this study and randomized into two treatment groups. Pre-treatment values for neuropathic pain, balance, and fall risk were evaluated before treatment. Post-treatment values for neuropathic pain, balance, and fall risk were recorded after the end of the sixth week. All participants received a total of 18 treatment sessions thrice a week over six weeks lasting for fifty minutes.

### Outcome Measures:

DN4 scale is used to evaluate the stage of neuropathy pain. It has ten questions, each question is evaluated on one to ten. If the point is greater than four it means this is neuropathic pain, if points are less than four then it means this is not neuropathic pain at all.^17^ BBS is used to determine balance performance and risk of falls. Fourteen items on the BBS scale, each scale has an ordinal score (zero to four). The total score ranges from zero (lowest balance stability) to fifty-six (highest balance stability).^18^ MFES is used to evaluate effectiveness while performing daily living activities without falling. Higher scores reflect less fear of falling, and more confidence.^19^

The TUG test was used to investigate mobility in adults. In this test, participants stand up and walk three meters (ten feet) and again turn back and sit on the chair and record time. If participants perform this test in less than ten secs then it means they have good mobility, and if greater than thirty seconds that means a high risk of falling. The range (seconds) of this test was according to age groups.^20^

Data Analysis was done using SPSS 25. The homogeneity test was applied at baseline. The normality test, the Shapiro-Wilk Test, assessed the normal distribution. The parametric and non-parametric tests were applied accordingly. Independent t-tests and paired t-tests are used in parametric tests, while Mann-Whitney U tests and Wilcoxon tests are used in non-parametric tests.

## RESULTS

**Fig.1:**
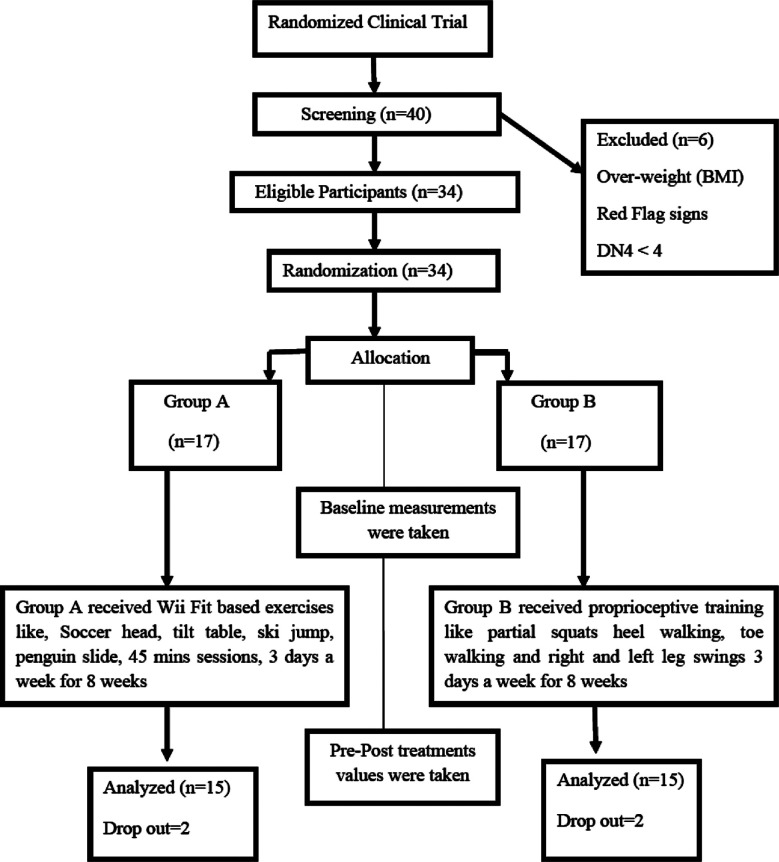
Consort flow diagram.

The data was analyzed using SPSS, IBM version 25. Both groups were homogeneous at baseline p>0.05 (independent t-test). The normality of the data was assessed by the Shapiro-Wilk test and parametric tests were applied accordingly. Four patients dropped out of the study due to some personal reasons (two participants had to go out of the city and two participants were reluctant to continue the sessions), two from Group-A, and two from Group-B (participant’s flow chart is shown in Fig.1). Analysis of thirty participants (20 males and 14 females) was done. An independent sample t-test was applied to determine any significant difference between the two groups. Paired sample t-test was applied to determine the within-Group-Analysis. A p-value less than 0.05 was considered significant.

The comparison of both treatment groups for the demographic elements is shown in Table-I. Group-A had 11 males and six females with mean body mass index (BMI), 27.92±2.646 kg/m^2^ while in Group-B there were nine males and eight females with mean BMI, 26.67±1.447 kg/m^2^. Between the Group-Analysis (Independent sample t-test) showed that both groups have no significant results in all outcome measures (p>0.05), which indicates there was no difference between both types of treatments shown in Table-II. But within the Group-Analysis (paired sample t-test) showed that both groups have significant results in all outcome measures (p<0.05), which indicates both types of treatments are clinically effective for the diabetic neuropathic patient shown in Table-II.

**Table-I:** Baseline Demographics of Both Groups.

Baseline Characteristics	Group-A	Group-B
N	17	17
Mean Age	60±9.016	63±9.342
Mean BMI	27.92±2.646	29±3.519
Mini-Mental State Exam	26.40±1.724	26.67±1.447
Gender	Males=11 Females=6	Males=9 Females=8

***Abbreviations:*** N=Total number; BMI=Body Mass Index.

**Table-II:** Across and Within-group comparisons of outcome measures of Group-A and B.

Outcome Measures	Group-A	Group-B	Mean difference	P-value
Pre-DN4	5.93±1.09	6.53±1.60	0.6	0.24
Post-DN4	5.93±1.09	6.53±1.60	0.6	0.24

	***Group-A***	***Group-B***	***Mean difference***	***P-value***

Pre. BBS	39.73±6.25	38.73±6.89	1.00	0.22
Post. BBS	45.80±5.79	42.60±5.52	3.20	0.13
Mean Difference	6.07	3.87		
P-value	0.04	0.00		

	***Group-A***	***Group-B***	***Mean difference***	***P-value***

Pre. TUG	22.20±3.00	22.60±3.33	0.40	0.60
Post. TUG	17.60±3.15	18.73±3.15	1.13	0.07
Mean difference	4.60	3.87		
P-value	0.00	0.00		

	***Group-A***	***Group-B***	***Mean Difference***	***P-value***

Pre. MFES	7.33±1.29	7.16±2.06	0.17	0.28
Post. MFES	8.73±1.09	8.46±0.88	0.27	0.21
Mean Difference	1.4	1.3		
P-value	0.03	0.04		

***Abbreviations:*** DN4=Douleur Neuropathique 4 Questionnaire; BBS=Berg Balance Scale; TUG=Timed Up And Go; MFES=Modified Fall Efficacy Scale.

## DISCUSSION

The purpose was to compare the effects of Wii fit-based exercises and proprioceptive training for improving balance and decreasing the risk of falls in diabetic neuropathic patients. There was no significant difference in both groups but clinically both groups show significant results (within Group-Analysis). The results show that both treatments were effective and showed clinical results in improving balance and reducing the risk of falls by using the assessment tools BBS, TUGT and MFES.

In a previous study, Wii Fit exercises effect was seen on the life quality, balance, and confidence of diabetic patients. The experimental group significantly showed improvement in balance, and quality of life in patients with diabetes (p≤0.05) as compared to the other group^9^ while in the current study, there was no difference in both groups as both groups were experimental and both treatments were effective clinically. The effects of exergames exercises on posture problems in diabetic patients were seen in a study and revealed significant improvement as compared to another group.^14^ Wii Fit is designed as a healthy interactive training for the public. In a clinical setting, Wii Fit is utilized for treatment to improve balance and posture. A systematic review was conducted to evaluate the contribution of Wii Fit in fields of a healthy population, encouraging fitness. In this systematic review, 115 articles included that Wii Fit is used for healthy and pathological problems, in some studies include that Wii Fit showed great improvement in chronic diseases.^21^

The performance of video games related to functional endurance, mood, and quality of life in older adults was investigated and worked better than exercise, especially for walking and balancing parameters.^22^ The current study also shows improvement in both groups but no significant difference in comparing across the group difference. In 2015, the first time, the Nintendo Wii balance board was used in clinical setup, this intervention was applied to patients with spinal cord injury. Tracy Wall conducted this study to improve daily life activities, posture/balance problems and reduced the chances of falls.

This intervention showed better results in posture and reduced balance problems in patients with spinal cord injuries.^23^ Wii Fit improved the postural sway and reduced falling in older adults in comparison with conventional physical therapy used in the six weeks study.^12^,^13^ In a quasi experimental study 2021, biodex balance system was used for the balance in diabetic neuropathic patients and it showed significant results. The balance was improved as in comparison with the current study results.^24^ If we compare the results of previous studies, it is concluded that Wii Fit is effective in improving balance and more challenging than any other exercise. The results varies due to the choice of comparison groups but in overall in diabetic neuropathic patients balance can be improved with virtual exercises.

### Limitations & Recommendations:

There was no follow-up considered due to lack of time and resources. Wii Fit is a cost-effective and user-friendly balance training tool popular nowadays. It is recommended that Wii Fit should be used in clinical rehabilitation in future for diabetic neuropathic.

## CONCLUSION

The study concluded that there was no difference between Wii Fit exercises and proprioceptive training in improving balance and reducing the risk of falls. However, both treatments showed clinical improvement in balance and minimize the risk of falls in diabetic neuropathy.

### Author’s Contributions:

**SSR, DH, Maryam I & Mehwish I:** Conceived, designed and did statistical analysis & editing of manuscript, are responsible for integrity of research.

**Maryam I & Mehwish I:** Literature search, Did data collection and manuscript writing.

**SSR and DH:** Did review and final approval of manuscript.
